# Eye tracking in Educational Science: Theoretical frameworks and research agendas.

**DOI:** 10.16910/jemr.10.1.3

**Published:** 2017-02-04

**Authors:** Jarodzka Halszka, Kenneth Holmqvist, Hans Gruber

**Affiliations:** Open University of the Netherlands, Netherlands; Lund University, Sweden; UPSET, NWU Vaal, South Africa; University of Regensburg, Germany; Turku University, Finland

**Keywords:** applied eye tracking, education, learning, expertise

## Abstract

Eye tracking is increasingly being used in Educational Science and so has the interest of the eye tracking community grown in this topic. In this paper we briefly introduce the discipline of Educational Science and why it might be interesting to couple it with eye tracking research. We then introduce three major research areas in Educational Science that have already successfully used eye tracking: First, eye tracking has been used to improve the instructional design of computer-based learning and testing environments, often using hyper- or multimedia. Second, eye tracking has shed light on expertise and its development in visual domains, such as chess or medicine. Third, eye tracking has recently been also used to promote visual expertise by means of eye movement modeling examples. We outline the main educational theories for these research areas and indicate where further eye tracking research is needed to expand them.

## Introduction

Eye tracking has been developed to measure ‘where we look at’. For a long time and up until now, optimizing the apparatuses to measure accurately and unobtrusively how the eyes move, considerations which eye movements can be distinguished from a neurological perspective (cf. the discussion of whether post-saccadic oscillations are separate eye movements or belong to saccades), and developing software to detect these different types of eye movements were in focus. These topics are still ongoing and there is still plenty room for this fundamental eye tracking research. But already from the beginning, these apparatuses were used – irrespective of the many fundamental unknowns and imperfections – to apply them to answer research questions from other fields. This applied eye tracking research field began with letting people view art paintings [93]. Quickly linguistics jumped onto the eye tracking train and this became probably the best investigated field of applied eye tracking research [67, 68]. Later on, usability and human-computer-interaction researchers discovered the value of eye tracking for their purposes [33]. A rather young field of applied eye tracking research is the one of Educational Science that we would like to introduce here to the readers. Let us begin with what Educational Science actually entails.

Educational Science investigates how people learn and how this learning can be fostered with instruction. But what is learning? Kids at school begin with deciphering single letters and end up analyzing complex texts and relate these to accompanying graphs or pictures. University students begin with studying countless facts over years to finally become highly specialized experts who effortlessly diagnose complex problems. Hence, learning is the act of acquiring or improving knowledge, skills or behavior. Its result is a persistent change of these. Learning follows a trajectory from an initial encounter with a topic or task, such as studying a textbook page for 30 minutes, to mastering it on high levels of expertise, in professional development lasting for decades. Thus, learning is rather a process than merely an outcome, such as a grade or a diploma. Researchers in Educational Science investigate this process to understand how learning is constituted and how it can be fostered through instruction.

**Figure 1:**
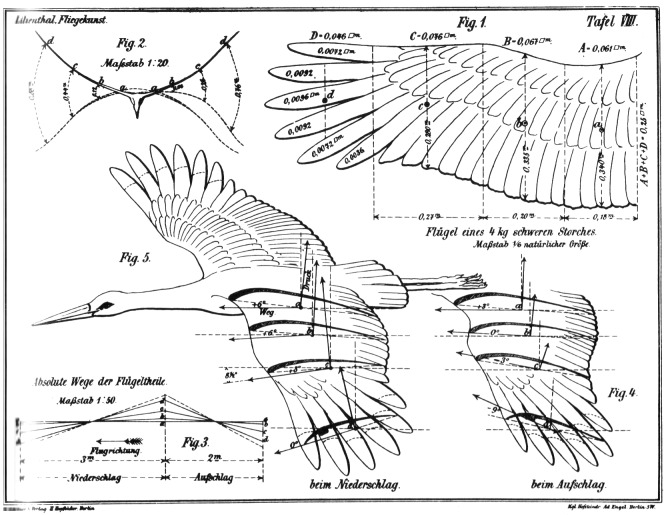
Scientific illustration on the flight of birds. Otto Lilienthal, Der Vogelflug als Grundlage der Fliegekunst, Berlin, 1889.

Eye tracking [29] has become an important tool to investigate learning processes over the past years. The reason for this is that we take most information in via our eyes; this is true when we learn, but also when we execute a professional task. Consider for instance scientific illustrations. Such illustrations on the composition or functioning of diverse systems have been around since hundreds of years. Below you see an example from the 19th century [48] on the flight of birds (Figure 1). Not only professionals had to deal with such illustrations, but also students had to use them to study the subject matter. Nowadays, with increasing possibilities to create visualizations, their use, but also their variability has mushroomed. For instance, professionals have to operate complex computer-generated simulations (e.g., interactive 3D medical images), while students have to learn from all sorts of visualizations, such as videos, and often they have to integrate information from many sources. And these are just few examples of where eye tracking can aid in understanding and even improving learning and its instruction within Educational Science.

Nowadays, learning often takes place in environments that are rich in information. These environments may be learning materials, such as textbooks or e-learning settings. But they may also be working environments, such as a surgical room for medical residents or a flight simulator for pilots. Often, they can be so information-rich that they can easily overwhelm the learner. Basically, there are two possibilities to deal with this issue. First, the environment can be adapted to the learner. This approach is most effective for initial stages of learning and is called Instructional Design. Instructional material that is designed to optimally make use of the human cognitive information processing system as well as the abilities of the learner enables the learner to autonomously and efficiently make progress. In later stages of learning, it is important to encounter the environments in their full complexity. This is for instance the case in workplace learning. In such cases, the second option comes into play, namely, scaffolding the learner to the environment. This part of educational research is called expertise development. Again with the long-term aim to enable the learner to autonomously develop. The theories used in Educational Science are based on findings from fundamental research on cognition and perception, but are at the same time applicable to concrete educational practice.

In the following we will describe these two areas of research in education with concrete examples from our own research. Next we will show how both areas can be integrated into a training method of visual expertise, called eye movement modeling examples.

## Instructional Design – adapting the environment to the learner’s abilities

### Theories of human learning – the working memory perspective

Let us begin with the initial stage of learning: a person who has little prior knowledge on a topic wants to learn new facts from a textbook, for instance about the functioning of a car engine. The material presented in this book contains a text describing the functioning of this engine, but also several graphs that show how the different elements of the engine would move at different stages of the stroke cycle. This person might experience quite some difficulties to relate all this information into one coherent mental model in his or her mind. He or she might be also distracted by a picture of a fancy car placed on this page. The research area of Instructional Design investigates how to construct learning material that optimally supports the learner. One very important aspect of this is how the material is visually presented.

The strongest focus in Instructional Design lies on the (visual) flow and processing of information to and within working memory. This view is based on (a simple version of) Baddeley’s working memory model [3] and Paivio’s dual coding theory [63]. The two most influential theories on Instructional Design are the Cognitive Theory of Multimedia Learning [54] and the Cognitive Load Theory [11]. Both theories assume that (a) the working memory capacity is limited and learning can only take place if enough capacity is available and not consumed by ‘bad’ Instructional Design. Moreover, (b) learning only takes place if the learner actively engages with the learning material or the task. The Cognitive Load Theory [11] mainly states that the working memory capacity can be consumed by different types of load that can either be attributed to the difficulty of the task itself (known as intrinsic load), ineffective layout of the instructional material (known as extraneous load), or active elaborations on the task content (known as germane load). Only the latter results in learning. The Cognitive Theory of Multimedia Learning [54] focuses on the working memory’s dual coding in interaction with the instructional material and long-term memory. This theory predicts how pictures and words are processed in working memory depending on their modality (written or spoken) and integrated with long-term memory content. For learning to occur, relevant information from the material must be visually selected and integrated, organized in mental models and integrated with prior knowledge. If this happens, a person learned. It is easy to see that the theories include statements on perceptual processes (e.g., visual search of relevant information; integration of information from different sources), although these processes were not directly tested when these theories were formed.

Both theories result in astonishingly similar guidelines on how to design (the layout of) instructional material [55, 81]. The aim of these guidelines is to decrease unnecessary cognitive processes (i.e., extraneous load) and to foster cognitive processes leading to learning (i.e., germane load). These guidelines shall make learning efficient (i.e., as much content learnt within as little time as possible). The most established guidelines include

Seeking **coherence of information**. First and foremost it is crucial to avoid unnecessary information presented in instructional material, such as decorative pictures. As the learner tries to make sense out of every information given and integrate it with the other presented information and with own prior knowledge, irrelevant information will only unnecessarily consume cognitive capacities.

Avoiding **redundant information**. The exact same information should not be given in different formats, because the learner tries to integrate all information with each other as well as with prior knowledge. This in turn costs cognitive capacities, which are not available for learning any more. One common ‘bad’ example is presenting a text on the slides and reading it out loud at the same time.

Making use of **multimedia**. Even though the exact same information should not be presented in different modalities, preferable the same subject matter should be presented in different ways. For instance, an explanation of a car engine is easier to understand with an accompanying picture or animation.

Making use of **different modalities**. To account for the dual-coding characteristics of working memory, instructional material should present related information in different modalities. For instance, a graph accompanied by an audio text instead of a written text.

Avoiding **split attention** by seeking contiguity. Instructional material should present related information that needs to be integrated in closely, both in space and time. For instance, the legend of a graph should better be incorporated in the graph itself than presented on the side.

More principles were developed over time and fill entire textbooks [54], but these are the most fundamental ones. These guidelines sound valid and were often supported by empirical studies – but not always.

### Testing learning theories in educational practice

The above described theories were developed based on many empirical studies that were conducted under specific circumstances. We will exemplify this with the studies of Mayer (for an overview of 15 years of studies: [54]) and describe how new studies should enrich these findings. First, most studies were conducted with psychology students as participants. This is common research practice, as psychology students have to participate in research for course credits and form the backbone of a lot of psychological research. For many research topics that should be equal across humans (e.g., perception, memory) psychology students are valid participants. For educational research, however, they represent a preselected group with very specific characteristics that may influence the outcomes (e.g., in Germany only students with very high grades are allowed to enter psychology study). Thus, we argue that it is crucial to test the actual target group of a learning material when investigating educational principles. Second, the illustrations used were very specific. Mayer used in most studies short black and white drawings (animated or static) showing the formation of lightning (or a bicycle pump). Of course it was important to keep the material constant when investigating different principles. Nowadays, however, we must acknowledge that this was a very specific format (simple black and white drawings) and a specific topic (shouldn’t lighting formation be known to university students?). Third, these studies used short, one sentence texts in English. This may have caused artefacts in the findings. For instance, research suggests that a modality effect only occurs for short sentences, while for long sentences only the last part is affected [73] or that it might even occur only for English text [50]. We argue that it is necessary to test the guidelines and principles found thus far on diverse material that probably uses more up-to-date multimedia.

**Figure 2:**
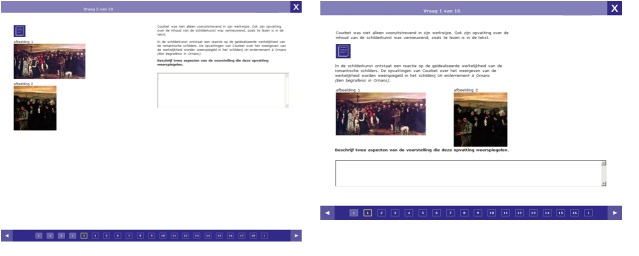
The computer-based testing environment in a split (left) and in an integrated design (right). Adapted from Jarodzka, Janssen, et al. (2015)[37].

In the following, we present two examples, where eye tracking shed light on the processes underlying these effects that were carried out in ecologically valid scenarios. In the first example, we tested the **split-attention effect** [37]. In our study, we used multimedia material on the topic of arts that is used nation-wide for assessment of all Dutch pupils at secondary school level. Moreover, our participants were 16 years old pupils. So, we used ecologically valid material that was tested with the actual target group. The material itself consisted not only of one task, but of eight tasks. Each task consisted of a text paragraph describing the task background and additional multimedia material, such as pictures, text or videos. We compared two versions of this material (Figure 2): In one version, all additional material was presented on one side of the screen and the task text on the other. This is a classic split-attention design as the pupils must visually search for the related information. In the second version, all additional material was placed within the text, right where it was referred to. This corresponds to a classic integrated design as it allows the pupils to process the multimedia information right when it is needed.

Surprisingly, pupils achieved better test scores in the split-version of the test (50% correct, vs. 44% correct in the integrated format). Eye tracking data showed that pupils largely neglected the additional information in the split-design (32 sec fixation time). Contrary to the predictions of the CTMML [54], pupils did not put a lot of effort to integrate the related information that would have consumed up cognitive capacity (5 points on a 9-point score for both conditions). Actually, these pupils were ‘lazy’ (or clever!) and ignored everything that they figured was not mandatory to solve the task. This was indeed the better strategy as it turned out that this additional information was not crucial to solve these tasks correctly. So was the integrated design pointless? On the contrary! Eye tracking results showed that exactly the same pupils processed all information in the integrated design (44 sec fixation time). Hence, they might have built a richer mental model in these cases. Probably, the test items were just not appropriately designed to tackle this richness of the mental model. Either way, learners might not always be as eager to actively process all given information as multimedia theories assume them to be.

**Figure 3:**
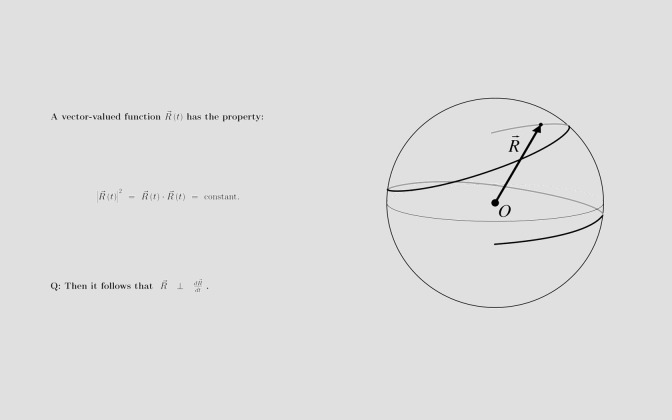
Exemplary task from the multimedia condition. Adapted from Ögren et al. (2016)[61].

In another example, we investigated the **multimedia effect** [61]. In this study, we used multimedia material on the topic of vector calculus. Again, for our participants this was relevant educational material, as they were university physic students. These students solved eight tasks. Each task was composed of a text describing the problem including a formula, and a statement about this formula that the students had to confirm or reject (i.e., task performance). Additionally, half of the problems included a graph that presented one exemplary instance of the formula (Figure 3).

CTMML would predict that such an additional visualization should enrich the mental model the students are building and thus, lead to better performance. This was not what we found (56% correct with graphs vs. 52% correct without graphs). Instead, we found a bias in students to confirm the statement, if a graph was present (65% confirmation vs. 47% rejection). This is in line with findings that scientific pictures make text appear more credible [56]. Hence, our students probably saw the graph, judged it as being correct and concluded the same for the statement. Eye tracking data revealed that in the multimedia tasks, students paid less attention to the task description (50% vs. 40%) and to the statement (45% vs. 40%) – obviously, as they also looked at the graph (20%). The amount of looking at the graph was not related to task performance (dwelling on graph when answering correctly 20% vs. incorrectly 19%). Looking at the statement, however, was positively related to task performance (dwelling on statement when answering correctly 43% vs. incorrectly 38%). Also, many transitions between the statement and the graph were positively related to task performance (correct answer: 9 transitions vs. 6 transitions for incorrect answers). Lin and Lin [49] received similar findings when investigating geometrical problem solving with eye tracking: while looking at the graph was an indicator for perceived difficulty, looking at the area where the task performance actively takes place (here: calculation area) was positively correlated with task performance. Consequently, we must specify the CTMML based on our findings: it is not enough that the learners process a graph; they must process it in the context of the main task question. Only then graphs are beneficial, otherwise they might even pursue learners to be uncritical. Moreover, a recent study by Krejtz, Duchowski, Krejtz, Kopacz, and Chrzastowski-Wachtel [44] has shown that the type of graph that is presented plays a role: interactive graphs evoke most systematic text-graph integrative saccades than static or dynamic graphs. Future research should investigate, whether this has also a positive effect on learning outcomes.

### Research agenda for Instructional Design theories

We can conclude from these two examples already that eye tracking can help to explain unexpected findings, as it allows unique insights into processes underlying learning outcomes. One possible reason for the unexpected findings might be that the perceptual processes assumed by multimedia theories (CTML, CLT) were not directly tested with eye tracking when these theories were developed. These theories have been very helpful heuristics to design instructional material. However, now we must unravel new evidence to further develop, specify, correct, and form these theories. The following issues should be considered in future eye tracking research to achieve this:

In the latter example presented above [61], we saw that the guidelines given, might need to be specified. Hence, it is crucial to test also the **other guidelines for Instructional Design** with eye tracking, but also under ecologically valid circumstances (i.e., actual learning material with real students).

In the first example above [37], we saw that even if assuming that those guidelines are appropriate, some **basic pre-assumptions of these theories** might not be (e.g., that learners do their best to actively integrate material). Hence, it is crucial to test also these. In particular, the many assumptions about perceptual processes must be tested directly with eye tracking.

The research discussed thus far considered cognitive processes. However, **metacognitive processes** are also crucial for learning (i.e., monitoring what I already can do what I still need to practice). However, too little research has been conducted on this important topic until now [84].

Finally, eye tacking research is conducted in laboratories where one participant at a time is tested under minimal disturbance. This has, however, nothing to do with educational practice. From social psychology research, we know that performing a task in the presence of others might be inhibiting, but also facilitating [9]. Eye tracking research also shows effects of social presence on attention [62, 72] Hence, future eye tracking research should investigate **social effects** on processes of learning, for instance within so-called digital classrooms.

It has to be noted that eye tracking – in particular in methodological triangulation with other process data – cannot only be used to derive instructional guidelines, but also to concretely usability test concrete computer-based multimedia learning environments. For a comprehensive description on how to proceed in such a case, see Groner and Siegenthaler [26].

## Expertise development – scaffolding the learner to the environment

### Theories of human learning – the long-term memory perspective

So far, we have looked into initial learning processes. The more a person knows about a task or a domain, the more we must take the long-term memory into account as well. In the long-term memory all knowledge is stored and with increasing experience in a task it is reorganized. This **knowledge organization**, in turn, changes the deal for the working memory. It changes it to this extent that Ericsson and Kintsch [20] suggested the concept of long-term working memory. For instance, with increasing numerical skills, children do not have to memorize six digits separately, but can form two chunks of three digits each and thus increase their working memory capacity [59]. With ongoing mathematical education, children can even solve mathematical problems described in text form. They quickly see the crucial cues that indicate which type of formula should be used. Based on this info, they know which other information they have to search for in the text and which they can ignore to fill in the formula. Next they solve the formula and formulate a solution to the problem. This procedure describes an exemplary use of a schema [88]. Similar to a chunk, a schema is not only an efficient way to store information in long-term memory, but it also expands working memory: one entire schema functions as only one entity. Thus, plenty capacity is left over to collect new information to fill in the schema’s empty slots. If a schema includes a specific temporal order, such as visiting a restaurant (enter a restaurant, look for a table, order from menu, …), it is called a script [74]. Another form of knowledge organization is forming short-cuts within long chains of reasoning by encapsulating parts of it into entities that are only unfolded into its pieces if necessary [10, 75]. The more knowledge a person has in a task and the more efficient it is organized, the faster and more correct this person can execute this task. Until he or she eventually becomes an expert [19]. For certain professions, such as medicine, we already know so much from research that these short-cuts and organizations of knowledge can be described very specific [35]. In the current section, we specifically focus on visual expertise and what we know so far about its knowledge and skill organization.

The specific case of visual expertise.

Expertise is defined as a consistently superior performance on a specified set of **representative tasks** for a domain [22, 24]. This superiority is due to the above described efficient organization of large amounts of knowledge and skills in a domain. This efficient knowledge organization reflects in different aspects, depending on the task itself. One example is the above mentioned well documented cognitive chunking in chess [12, 15]. Typically, expert and novice chess players are asked to build chess formation from memory; a task in which experts excel largely [25, 27]. Eye tracking research revealed that this chunking is also reflected in perceptual processes: experts look rather in between chess figures, while novices look at each single figure [69, 70]. We see that the concept of chunking in chess is reflected in two aspects: a cognitive recall performance and perceptual processes. Similar findings occur also in other domains of expertise, such as playing music [47]. In most cases, reading from notes is an important part of playing music and thus, it is one aspect of musical expertise that is investigated with eye tracking [1, 64, 65, 66].

Reingold and Sheridan [70] provide a comprehensive overview of eye tracking research on visual expertise. The authors draw two main conclusions from their review. First, experts are able to encode domain related patterns in a superior way, which is due to their larger visual span. Second, eye tracking data of experts often entails information that they were not aware of. This is a clear indicator of experts’ tacit knowledge. The increased visual span is a reflection of the above described chunking in perceptual processes. The tacit knowledge could be linked to encapsulated knowledge and its automated use. When reading this review, you will quickly realize that most research was conducted on the traditional expertise domains of chess and medicine. These studies used static and perceptually simple stimuli, such as chess boards or X-rays of the chest.

However, a lot of visual expertise plays a role in perceptually much more complex environments, such as air traffic control [7], new medical imaging techniques [8], meteorology [80], etc. These environments are difficult for cognitive processing for two reasons [2, 11, 54]. First, they are information-rich [18, 76]. Hence, they entail large amount of information; and a lot of it is irrelevant. On top of that, the relation of thematic relevance and visual saliency is often not optimal. Hence, it is challenging to select the relevant information. Moreover, these environments are dynamic [28, 52]. Thus, information may be transient. Also, several information elements may appear (and disappear) simultaneously (cf. split-attention effect). Consequently, it is challenging to keep information active so that it can be integrated. Consequently, the stimuli used in most visual expertise research so far are not representative for most expertise domains. Thus, we cannot simply generalize these findings to information-rich or even dynamic domains. Research in this field, is in focus of the following section.

### Research on visual expertise in information-rich environments

The concept of visual expertise is difficult to tackle as it entails so many different aspects (as already described above). In most cases, it is thus necessary to approach this concept from different angles by means of methodological triangulation [16, 82]. One the one hand, eye tracking can tackle the perceptual aspects of visual expertise, while other data sources complete the picture on the more cognitive side, such as performance data, verbal data, and even drawings of what a person thinks where he or she looked at. Due to the nature of this concept and the research tradition, verbal data are most often used to investigate expertise [23]. They can take the form of interviews, self-explanation, retrospective reports or thinking aloud (for an overview of different forms of verbal data and how to combine them with eye tracking see Chapters 3.4.8 and 4.7.3 in Holmqvist et al. [29]). If implemented carefully, verbal reports will not disturb the actual task performance. Instead, they will give us more information on the reason why a person looked at a certain area. In the following, we present examples from own research using this methodological triangulation for investigating visual expertise and its knowledge organization in information-rich environments.

One reason that this field is still so little investigated [70] besides its obvious relevance as described above, are software issues. In 2010, we published the very first article investigating visual expertise with eye tracking using video material and an AOI analysis [38]. This study investigated expertise in the domain of marine zoology. In other words, seven professors and PhD students, and 14 biology students classified the swimming modes of reef fish. In reality, marine zoologists often execute their profession under water (either snorkeling or diving). To get as close as possible to this situation, we asked participants to watch four videos of single fish swimming in a colorful reef for as long as they wanted to. In this way, we created representative, but at the same time experimentally controllable tasks. Afterwards, they watched their own eye tracking recordings and reported what they were thinking while approaching this task [87]. As we wanted to compare where experts and where novices looked at, we used a cumbersome manual procedure to define AIOs on videos, which delivered interesting findings: Experts clearly outperformed novices (experts: 4/4 points, novices: 3/4 points; η_p_^2^ =.18), which meant that they were indeed true experts in this task (not a trivial finding in expertise research!). Also, we compared the sequences in which participants inspected the different body parts of the fish. Experts were more diverse than novices (similarity of experts: 67%; novices: 72%; η_p_^2^ =.08). Probably, novices just followed the most visually salient features, which resulted in a rather similar scanpath. Experts, on the other hand, seem to have had different scripts to approach this task, which resulted in different scanpaths. These different scripts might be due to different forms of experience (i.e., when diving you see the fish from the side, while when snorkeling you see it from above; consequently, you rely on different features when classifying its motion). Indeed, dwell time analyses of AOI data taken together with participants’ verbal reports, showed that part of the experts took a short-cut: they first classified the fish and deduced from this, how it must swim (dwell time on according AOIs of experts: 375 ms; novices: 160 ms; η_p_^2^ =.29; according verbal utterances of experts: 57; novices: 26; η_p_^2^ =.56). In sum, we found that visual expertise in marine zoology (a) leads to different types of scripts, probably depending on the concrete experience in that task, and (b) which form these scripts can take.

In a following step, we moved towards an interactive task stimulus, namely digital pathology [30, 31, 32]. In the first study [31] we compared how participants of three expertise levels diagnosed 10 pathological slides based on a two seconds inspection. They were eye tracked during this inspection and reported afterwards how they came about their diagnosis. Obviously, novices were incorrect (38% correct diagnoses), incomplete and inconclusive in their diagnosis (hardly conclusive terms or diagnostic specifications mentioned) and looked little at relevant areas (3 fixations). Experts (85%) and intermediates (87% correct diagnoses), on the other hand, diagnosed these slides equally well. However, they differed in how they processed the slides. Experts relied on their first inspection of the relevant area (fixation dispersion 1st trial part: 135) and then further checked the slide for other potentially relevant information (2nd trial part: 167). In their explanations they mainly focused on the typicality of the slide (e.g., high usage of comparative terms). Intermediates kept inspecting the relevant area throughout the entire trial duration (fixation dispersion 1st trial part: 192; 2nd: 165) and considered many potential diagnoses (e.g., a lot of mentioning of pathologies). For their knowledge organization, we may conclude that experts have such consolidated illness-scripts that they can rely on, which leaves them capacity to check for further potential problems. Intermediates, instead, possess already according schemata, however, they still have to check many competing schemata to reach a diagnosis. Even though this study yielded interesting findings, the task we used was not really representative for this profession. Hence, in following studies [32, 30], we used a digital version of a tissue sample that could be operated as under a regular microscope: zooming in and out as well as panning around the slide. Hence, this was a highly representative task. Despite the progress in commercial eye tracking software, using a stimulus that can be individually changed that much (and that is not a website) is still challenging and requires a lot of manual work and programming. We found that experts were more efficient as they used fewer microscopic movements (e.g., opposed zooming movements: η2p = 0.03; expertise effect for all navigation behavior: η2p= 0.11) and shorter reasoning chains to reach a diagnosis (reasoning terms used by experts: 109; intermediates: 63; novices: 159). This is in line with the findings from the first study that indicated that experts possess consolidated illness-scripts that allow fast decision making. Also, navigation data showed that experts visited fewer diagnostically relevant areas (experts: 3.05; intermediates: 3.98; novices: 4.05). This poses the question whether it is even possible to define areas as being relevant for each expertise group. It might be difficult to grasp the effects, because experts understand the stimuli so quickly. Intermediates also showed processes that are in line with Study 1: they took longer to reach a decision (expert: 86 sec; intermediates: 110 sec; novices: 152 sec) and looked more at relevant areas while basing their diagnosis on many specific abnormalities (novices: 35; intermediates: 96; experts: 94). Thus, intermediates already have established schemata. However, they still need a lot of time to check them. Novices again were simply all over the place and clearly lacked any relevant knowledge (or its organization).

Another expertise domain we have investigated is air traffic control [36, 90]. Controlling air traffic is a really challenging task: constantly flying in and departing airplanes need to be coordinated with a high emphasis of safety, but also on environmentally friendly travel. 31 air traffic controllers of three different expertise levels solved nine situations. Each depicted a real radar screen, with airplanes (including type, height, and speed), sectors, and start and landing points. Participants reported the optimal order of arrival of the airplanes while their eye movements were recorded. Individuals with higher levels of expertise clearly outperformed those of lower levels (experts: 4.63, intermediates: 4.30; novices: 3.82; η_p_^2^ = .49). Interestingly, the performance of those with higher expertise was more similar than of those of lower expertise (experts: 0.59; intermediates: 0.53; novices: 0.43; η_p_^2^ = .44; in contrast to our findings with marine zoologists: Jarodzka et al. [38]). In this profession it seems, thus, that there is one optimal script to solve this task. Eye tracking analyses revealed that individuals with higher expertise looked mainly at the aircrafts and at the background between them (e.g., time to first fixation on aircraft for experts: 41.59 sec; intermediates: 54.6 sec; novices: 65.06; η_p_^2^ = .37). This indicates that the script individuals with more expertise establish allows them to better focus on the relevant information and chunk single information entities. Novices, on the other hand, had no appropriate strategy to relay on and fall back on the sub-optimal means-end-strategy as indicated by them looking mainly at the destination of the airplanes (e.g., time to first fixation on destination for experts: 38.38 sec; intermediates: 36.62 sec; novices: 25.37 sec; η_p_^2^ = .36). We have to admit, though, that participants only saw static screenshots of radar screens. In a recent follow up study, we used a more representative task of this profession [36]. In that twelve participants with varying expertise levels worked on a simulation of an actual airport. The situation was entirely realistic including communication with other co-workers. Already the first eye tracking analyses reveal a drastic difference to the first study: novices mainly focus on the area of their own responsibility, while individuals with higher expertise look more outside this area, including the starting and landing points of the planes. This strategy allowed them to plan ahead in this very dynamic environment. Hence, the scripts individuals with higher expertise possess in this task, must be updated dynamically if the task includes more time pressure.

### Research agenda for visual expertise research

From the research presented above, but also from other research on visual expertise of teachers [45, 92], neurological pediatrists [4], or radiology [43, 83] we have learned already a lot about visual expertise in information-rich environments. Experts use chunks (e.g., air traffic control) and shortcuts (e.g., marine zoology) and this can be also seen in their perceptual processes and measured with eye tracking. Also, we have clearly seen the use of cognitive scripts or schemata and their influence on the visual processing of an environment and vice versa in each profession. Often, even very concrete statements about the form of these schemata or scripts could be made. Still, many open research questions remain.

To which extent can we generalize these findings? We have seen that sometimes even slight changes in the task can lead to different outcomes (cf. air traffic control), while sometimes the changes go in the same direction (cf. pathology). Also, some findings that are found in one profession (e.g., experts become more similar in air traffic control) are not true for another profession (e.g., experts in marine zoology become more diverse). Hence, future research should consistently vary task characteristics and professions to understand, which aspects of visual expertise are generic and which domain-specific.

A lot of research on visual expertise has been conducted on simplified tasks. This was largely due to technological restrictions of the eye tracking apparatuses and software. Research should not be hold back by technological obstacles, but rather feed their development. In particular two issues must be tackled to foster ecologically valid research on visual expertise. First, the detection of smooth pursuit to enable valid analysis of dynamic stimuli. Thereby, it is not enough to detect smooth pursuit with a stand-alone algorithm, but it must be implemented into existing analysis software, so it can be used in applied research as well. Second, more automated analyses for mobile eye tracking. Clearly, the truest way of analyzing visual expertise often requires real-world eye tracking. However, cumbersome manual analyses often hold researchers back.

The presented research has shown how much we can benefit from methodological triangulation when investigating multifaceted concepts such as visual expertise. In a next step, research should directly link the analysis of verbal and eye tracking data. Only in this way it will be possible to make more concrete statements about the cognitive structures underlying these processes.

Finally, it must become the ultimate aim of this research line to unravel the organization of knowledge and skills in long-term memory and how it develops with increasing expertise. Only then it is possible to draw meaningful conclusions from eye tracking data that go beyond superficial statements such as ‘experts had longer fixation durations’ that have virtually no meaning for professional or educational practice [42].

## Eye movement modeling examples: Bridging Instructional Design and expertise research

### Theories of human learning – training visual aspects of expertise

So far, we have discussed how initial learning takes place, how it can be supported by Instructional Design, and which role eye tracking can play in this. Then, we have shown how individuals develop further over time and until they become experts in visual domains. In this section, we try to bring both research areas together to show how this road to visual expertise can be supported by instruction. This is not as trivial as it may sound, as Instructional Design entails the simplification of learning material, while expertise development requires to be faced with the authentic, information-rich tasks.

One very powerful way of learning authentic tasks is imitation. It is so inherent to our system that even two weeks old babies imitate adults [58]. Bandura [5] has shown in his classic bobo doll experiment that imitation leads indeed to learning. Children watched videos of an adult playing with a ‘bobo doll’, which is an inflatable, large doll that stands up again once it is tipped over. Depending on the experimental condition this adult was either behaving aggressively (e.g., punching the doll) towards this doll or not. Once these children were confronted with this doll, they treated it in a similar way as the model they saw in the video before [6].

Consequently, research on teaching and training has picked up this approach. Indeed, decades of research have shown that studying examples of a model successfully executing a task is more efficient for learning than learning by trial-and-error [41]. It is not trivial, though, to model a task. Many critical processes are not observable from outside, such as solving a mathematical equation. In such cases the model verbalizes his or her thoughts (cognitive apprenticeship: Collins, Brown and Newman [14]); process-oriented modeling examples: Van Gog, Paas and Van Merrieboer [86]). But what about perceptual processes in a visual task? We know that simply telling beginners to “look the way experts do” does work, but does not necessarily improve their performance [43]. These beginners may now know where to look, but not why.

**Figure 4:**
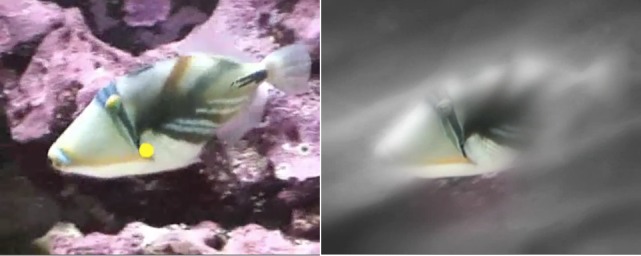
Eye movement modeling examples with a traditional dot display (left) and a spotlight display (right). Material used in Jarodzka et al. (2010)[38].

To address this issue, we developed eye movement modeling examples (EMME). These are video recordings of a model executing a task and explaining how he or she goes about that. On top of that, the model’s eye movements are tracked and replayed on top of the video [85]. However, novices are often already overwhelmed with information-rich material that forms the basis of visual tasks. Adding an eye movement display on top of that, is likely to overwhelm them. An alternative is to display the model’s eye movements by reducing existing information on videos [17, 60]. This results in a spotlight wandering across the video, while the rest of it appears blurred. Figure 4 presents screenshots of both, a traditional and a spotlight display used in EMME.

### Research on eye movement modeling examples

Research described in the last section has shown that experts dramatically differ from novices. Hence, there is no point in trying to ‘make novices act like experts’. Consequently, in our research, we have always used a systematic way to make the expert model act more didactical. On the one hand, the models in our studies were always not only experts in their domains, but also highly experienced in teaching this domain. Hence, they knew from experience which difficulties students face in these tasks and how to best explain these tasks to them. On the other hand, we used a specific recording procedure to ensure that the EMME videos were of high quality. First, to ensure a close relation of the voice and the eye movements of the models, we first show them the task itself (e.g., a video recording of something they need to classify). Only after they are familiar with this specific task, we begin with the recording. Such recording procedure have resulted tight gaze-voice couplings elsewhere [71]. Second, to shift the models’ focus from the task to the novice recipient, they evaluate their own recordings based on several questions: Will a student know what each term means? Is the task explained in comprehensible enough terms for students? Is it explained in enough detail? Are all information that a student needs contained? Are all contained information really important? Such questions have shown to improve written communication of experts to novices [40]. Third, if necessary, the models could revise their recordings.

We have used such EMMEs, for instance, to train the classification of the locomotion patterns of reef fish [39]. In the learning phase, participants studied four videos with either a dot display EMME, a spotlight EMME or a video with verbal explanations only (Figure 4). In the meantime, their eye movements were recorded to study whether they actually did follow the eye movement display of the model on the videos. In the testing phase, participants watched four new videos without any form of guidance or verbal explanation. They had the task to classify these videos accordingly. While watching the testing videos, participants’ eye movements were recoded to investigate the efficiency of their visual search of relevant information on the videos. Then, they indicated via a questionnaire how they interpreted this information. Results showed that both EMME videos guided the eye movements of the participants to the spots where the model looked at (measured as coherence between the model’s and the learner’s scanpath: Spot = 15.10; Dot = 15.11; Control = 12.07; η_p_^2^ = .39). Moreover, in the spotlight condition, participants showed a more efficient visual search on testing videos (measured e.g., time to first fixation on relevant areas: Spot = 1236 ms; Dot = 1530 ms; Control = 1632 ms; η_p_^2^ = .11), while participants in the dot group exhibited better interpretation performance in comparison to the control group (measured as % correct: Dot = 74%; Spot = 69%; Control = 67%; η_p_^2^ = .12).

We have conducted a similar study in the domain of diagnosing epileptic seizures in infants [34]. The experimental procedure was just as in the study described above, except from the task: participants watched videos of infants either suffering a form of epileptic seizure or a differential diagnosis. Even though both tasks sound very different, they had crucial commonalities: participants had to identify relevant body parts (fins that were used to produce propulsion vs. limbs that might be affected by the disease) and to describe how exactly these body parts move. Based on these two steps, a classification or a diagnosis, respectively, can be made. Also, these steps rely on a visual inspection of a video input. A further difference to the fish locomotion study was that display of the eye movements: The traditional display was shown as a circle instead of a dot to not occlude relevant information on the video (e.g., a twitching eye). The spotlight display was far more subtle than in the fish locomotion study. Results showed an overall advantage of the spotlight display on attention guidance in the learning phase (measured as Euclidean distance to model’s gaze: Spot = 210; Circle = 238; Control = 237; η_p_^2^ = .13), visual search (measured as e.g., time until looking at relevant area: Spot = 189 ms; Circle = 274 ms; Control = 289 ms; η_p_^2^ = .13) and interpretation performance (measured as % correct: Spot = 60% Circle = 53%; Control = 50%; η_p_^2^ = .11) in the testing phase.

Similar training approaches have been used for visual tasks, which require hardly prior knowledge [51, 53, 77], for expertise tasks [46, 57, 79], and even for problem solving in dyads [13]. However, these studies did not test whether the found performance differences could be transferred to similar tasks (as we did on our studies), i.e., whether learning took place. Thus, strictly speaking, these cannot be seen as educational studies.

### Research agenda for EMME

EMME as similar gaze-based approaches may be helpful in training visual tasks. Still, we should not become too enthusiastic, as there are also enough examples where these approaches had no (single conditions in the two studies reported above) or even detrimental effects [78, 85]. Hence, the question is not whether EMME does foster the performance of visual tasks (or even visual expertise), but rather, under which circumstances in does so. We thus recommend the following research questions to be addressed in the future:

The role of the task and the stimulus characteristics: The research on EMME covers a diversity of tasks (from insight problem solving, to performance only, to transfer and learning) and a diversity of stimuli (from simple line drawings to complex videos). A systematic variation and concrete description of these factors should shed more light into when EMME are effective. For instance, existing studies already indicate that the visual complexity of the task is crucial: Van Gog et al. [85] used a task that could be executed without perceptual input and found negative effects of EMME on performance [89]. Jarodzka et al. [39] used a fish locomotion classification task where all relevant information was visual salient. EMME was in part helpful in this case. Jarodzka, Balslev, et al. [34] used a pediatric neurology task, where the relevant information was transient and not salient. This is where EMME were most helpful.

The role of the eye movement display design is an entirely understudied aspect. Apart from two studies [34, 39], none has compared different designs directly even though these studies indicate that this might be a crucial success factor for EMME. Results showed that reducing information on a spotlight manner guides visual attention on EMME videos best. Also, the spotlight facilitates visual search on testing videos most. However, the interpretation of relevant features is only enhanced, if a holistic processing is possible during learning.

Moreover, the role of didactizing the expert model, as we have done in our studies, has not been directly investigated. In fact, most studies provide hardly any description on how the model’s eye movements were collected. This is surprising as we know very well from research to which large extent experts and novices differ in their processing and how unlikely it thus is that forcing experts’ processes upon novices can hardly work.

Finally, the EMME methodology could be embedded into well-established methods of expertise trainings. For instance, the 4C-ID training [91] is an elaborated model to design a curriculum for complex tasks. It includes modeling episodes that might easily be filled in with EMME for specific visual tasks. Another example is deliberate practice [21]. This method involves a detailed study of own and others performance. If the task includes visual aspects, studying the eye movements of an expert (or one owns) might provide additional benefits.

## Discussion

In the current paper we have introduced Educational Science as a field of applied eye tracking research. We have structured it along three topics, namely Instructional Design, expertise development, and eye movement modeling examples. The topic of Instructional Design investigates how learning of a new skill or knowledge by optimally designing the according learning material. Educational theories on human cognitive processing, in particular in the working memory, resulted in guidelines on how to design such material and which processes learners should devote to efficiently achieve learning gains. Up until now, eye tracking helped us to understand how learners actually process such instructional material, which was not always in line with what theory predicted. Future eye tracking research on this topic can thus help to further corroborate, improve, and enrich these theories. Not only to understand and support processes of initial learning, but also to better understand how we as humans process information in working memory under realistic circumstances.

The topic of expertise development investigates the other side of the learning spectrum, namely people, who already have a lot of experience and knowledge on a task. How do they process information? How do they differ from people with slightly less or more experience? A large body of expertise research started already many years ago to expend towards visual processes underlying expertise and thus, eye tracking research. This research showed that, indeed, changes in long-term memory structures that come along the development of expertise influence not only working memory processing, but also visual processing of the environment and vice versa. Future eye tracking research on this promising topic must dive into more real-world scenarios with diverse tasks and information-rich, dynamic environments. Not only will we understand in this way more about the development and characteristics of visual expertise, but we will also better understand how long-term memory structures influence the way we see and interpret our environment, both in every day and in challenging situations.

The third topic we have presented are eye movement modeling examples. This is the youngest topic within the field of applied eye tracking research in Educational Science, but nonetheless, a very promising one. It addresses the question, how visual expertise could be trained with the help of instructional videos of real-world tasks that are explained by experts in the field. These videos include an overlay of these experts’ visual focuses to support the learner in connecting the verbal explanation of the expert to the real-world complexity of the task. Of course, this research topic gives us practical implications for educational practice. But it also provides interesting research questions apart from education, such as: how to best guide eye movements of people on videos? How to support speech comprehension with displaying the eye movements of the speaker to the listener? Etc.

It is important to keep in mind that the area of applied eye tracking in Educational Science is clearly applied research. This means that the tasks and stimuli used are very diverse and less well controlled in comparison to fundamental experiments in vision science, for instance. However, they are ecologically valid. This is crucial for this research to allow drawing actual conclusions for educational practice. Therefore, research questions should always be developed together with stakeholders from educational practice. And the models or frameworks derived in research should always be tested ‘in the wild’ (aka schools, universities). But this also means that we can learn a lot from this research field on real-world processing, which in turn can be fruitful to establish new research question for fundamental research.

Furthermore, this research area is still relatively new. This means that there are no well-established eye tracking measures, like in reading research, that can be clearly related to concrete processes. This is due to the fact, that there is simply less research conducted as, for instance, in reading. But the ecologically valid nature makes it almost impossible to hope for such simple relations: each learning environment, each expertise domain are so inherently different in terms of tasks and stimuli that the eye tracking measures have to be found each time anew. The process of finding the appropriate measures must not be driven by what is given by the manufacturers. Instead, it is important to work along existing theories and carefully operationalize measures that are clearly related to concrete hypotheses.

## Acknowledgements and Conflict of Interest

This paper is based on two keynote speeches of the first author at the 4th Polish Eye Tracking Conference, Warsaw, Poland (2016) and the 7th Scandinavian Workshop on Applied Eye Tracking, Turku, Finland (2016).

The authors declare that there is no conflict of interest regarding the publication of this paper.
